# Association of Ficolin-3 with Severity and Outcome of Chronic Heart Failure

**DOI:** 10.1371/journal.pone.0060976

**Published:** 2013-04-15

**Authors:** Zoltán Prohászka, Lea Munthe-Fog, Thor Ueland, Timea Gombos, Arne Yndestad, Zsolt Förhécz, Mikkel-Ole Skjoedt, Zoltan Pozsonyi, Alice Gustavsen, Lívia Jánoskuti, István Karádi, Lars Gullestad, Christen P. Dahl, Erik T. Askevold, George Füst, Pål Aukrust, Tom E. Mollnes, Peter Garred

**Affiliations:** 1 IIIrd Department of Internal Medicine, Semmelweis University, and Research Group of Inflammation Biology and Immunogenomics, Hungarian Academy of Sciences, Budapest, Hungary; 2 Laboratory of Molecular Medicine, Department of Clinical Immunology, Section 7631, Rigshospitalet, Faculty of Health Sciences, University of Copenhagen, Denmark; 3 The Research Institute of Internal Medicine, Oslo University Hospital Rikshospitalet, Medical Faculty, University of Oslo, Norway; 4 Center for Heart Failure Research, Oslo University Hospital Rikshospitalet, Medical Faculty, University of Oslo, Norway; 5 Department of Immunology, Oslo University Hospital Rikshospitalet, Medical Faculty, University of Oslo, Norway; 6 Department of Cardiology, Oslo University Hospital Rikshospitalet, Medical Faculty, University of Oslo, Norway; 7 Section of Clinical Immunology and Infectious Diseases, Oslo University Hospital Rikshospitalet, Medical Faculty, University of Oslo, Norway; Oxford University, United Kingdom

## Abstract

**Background:**

Inflammatory mechanisms involving complement activation has been shown to take part in the pathophysiology of congestive heart failure, but the initiating mechanisms are unknown. We hypothesized that the main initiator molecules of the lectin complement pathway mannose-binding lectin (MBL), ficolin-2 and ficolin-3 were related to disease severity and outcome in chronic heart failure.

**Methods and Results:**

MBL, ficolin-2 and ficolin-3 plasma concentrations were determined in two consecutive cohorts comprising 190 patients from Hungary and 183 patients from Norway as well as controls. Disease severity and clinical parameters were determined at baseline, and all-cause mortality was registered after 5-years follow-up. In univariate analysis a low level of ficolin-3, but not that of MBL or ficolin-2, was significantly associated with advanced heart failure (New York Heart Association Class IV, p<0.001 for both cohorts) and showed inverse correlation with B- type natriuretic peptide (BNP) levels (r = −0.609, p<0.001 and r = −0.467, p<0.001, respectively). In multivariable Cox regression analysis, adjusted for age, gender and BNP, decreased plasma ficolin-3 was a significant predictor of mortality (HR 1.368, 95% CI 1.052–6.210; and HR 1.426, 95% CI 1.013–2.008, respectively). Low ficolin-3 levels were associated with increased complement activation product C3a and correspondingly decreased concentrations of complement factor C3.

**Conclusions:**

This study provides evidence for an association of low ficolin-3 levels with advanced heart failure. Concordant results from two cohorts show that low levels of ficolin-3 are associated with advanced heart failure and outcome. The decrease of ficolin-3 was associated with increased complement activation.

## Introduction

Chronic heart failure (CHF) is initiated by a variety of insults, including myocardial infarction and ischemic heart disease, hypertension, valvular abnormalities, as well as various forms of cardiomyopathies, which result in impaired myocardial function [1]. Following an initial impairment, there is an adaptive compensatory response to preserve the overall function. These compensatory responses may in turn eventually lead into maladaptive responses with development of progressive myocardial dysfunction and overt heart failure. Several studies have suggested the involvement of innate immunity and inflammation in these maladaptive responses, but at present, the underlying molecular mechanisms are only partly clarified [2].

The complement system represents an important branch of inflammation and innate immunity [3]. Complement activation occurs in CHF [4] and has been associated with adverse clinical events in patients with symptomatic heart failure [5], [6]. Deposition of the terminal complement complex (TCC) has been observed in myocardial biopsies from patients with dilated cardiomyopathy [7], but its relation to disease severity in cardiomyopathy is debated [8].

Activation of the complement system is mediated via three different routes: the classical-, the lectin- and the alternative pathways, which all lead to activation of the central complement component C3 and subsequently C5 with generation of C5a and TCC. In humans five recognition molecules of the lectin pathway have been described: mannose-binding lectin (MBL), ficolin-1 (M-ficolin), ficolin-2 (L-ficolin) and ficolin-3 (H-ficolin or Hakata antigen) and lately also collectin-11 [9], [10]. Ficolin-3 is the predominant plasma molecule with a median concentration in healthy Caucasians of 25 µg/ml, followed by ficolin-2 (5 µg/ml) and MBL (1 µg/ml), respectively [11]–[13]. By contrast, ficolin-1 and collectin-11 normally circulates at very low plasma levels [14], [15]. There appears to be a hierarchy in the complement activation potential between the lectin pathway initiators, as ficolin-3 has the strongest potential, MBL and ficolin-2 are intermediate, while ficolin-1 and collectin-11 may have the smallest potential [10], [16].

Because these molecules bind altered self and dying host cells we hypothesised that one of the underlying mechanisms of the observed complement activation seen in patients with heart failure could be due to involvement of lectin initiator molecules, reflected as possible consumption. Thus, we investigated the possible association between the main lectin pathway initiators MBL, ficolin-2 and ficolin-3 and clinical, hemodynamic and neurohormonal parameters of disease severity, as well as outcome in two independent prospectively designed cohorts of CHF patients originating from Hungary and Norway.

## Results

### Study Cohorts

One-hundred and ninety (Hungarian cohort, New York Heart Association (NYHA) functional class I-IV) and 183 (Norwegian cohort, NYHA II-IV) patients with stable CHF were consecutively included in the study (Table 1). The etiology of CHF was determined on the basis of disease history, coronary angiography and echocardiography. The main difference between the two cohorts was in the survival rates: 44% of the Hungarian and 69% of the Norwegian cohorts survived the 5 year long follow-up period. In addition, patients in the Hungarian cohort were older, with male predominance and had increased prevalence of ischemic heart disease as compared to the Norwegian cohort. Accordingly, concomitant atherosclerotic and related diseases such as hypertension, type-2 diabetes mellitus and previous myocardial infarction were more prevalent in the Hungarian cohort than in the Norwegian cohort.

**Table 1:** Baseline clinical characteristics in the two cohorts.

Variable	Hungarian cohort, n = 190	Norwegian cohort, n = 183
Age	69 (59–77)	58 (50–64)
Male (%)	141 (74)	143 (74)
Cause of CHF: CAD/IDCM/other (%)	108/41/41 (57/21.5/21.5)	76/96/8 (42/53/5)
NYHA Class I/II/III/IV (%)	38/62/67/23 (20/33/35/12)	0/51/79/49 (0/28.5/44/27.5)
Hypertension (%)	131 (69)	27 (15)
T2DM (%)	72 (38)	22 (12)
Previous infarction (%)	77 (41)	62 (34)
LVEF (%)	34 (27–40)	29 (20–40)
ACE-I or ARB (%)	130 (68)	155 (85)
Beta blocker (%)	128 (67)	146 (80)
Loop diuretics (%)	141 (74)	130 (71)
Statins (%)	75 (39)	79 (43)
Warfarin (%)	76 (40)	81 (45)
ASA (%)	72 (38)	78 (43)
Creatinine (µM/L)	96 (78–136)	89 (78–110)
CRP	6.2 (2.9–13.7)	3.6 (1.8–7.9)
NT-proBNP (pM/L)	680 (373–1561)	235 (92–497)

CAD: coronary artery disease; IDCM: idiopathic dilative cardiomyopathy; NYHA: New York Heart Association; T2DM: Type-2 diabetes mellitus; LVEF: Left-ventricular ejection fraction; CRP: C-reactive protein; NT-proBNP: N-terminal pro-brain natriuretic peptide; ACE-I: angiotensin converting enzyme inhibitor; ASA: Acetyl-salicylic acid; there were missing data in 3 and 4 cases (Norwegian cohort for etiology and NYHA class).

### Lectin Complement Pathway Components in CHF

**Figure 1:**
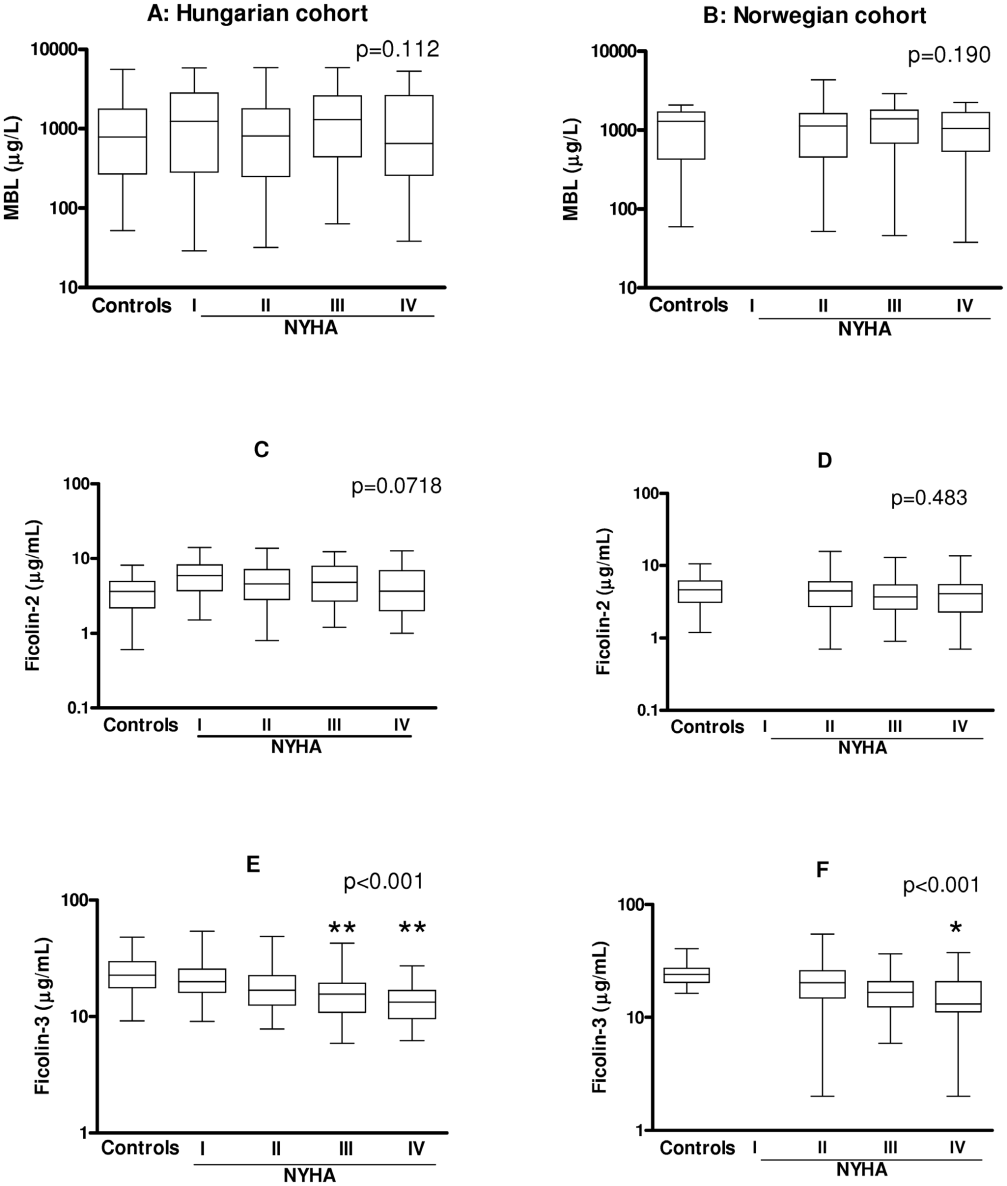
Association of MBL (panels A–B), ficolin-2 (C–D) and ficolin-3 (E–F) levels with severity of CHF. P values of non-parametric Kruskall-Wallis test are indicated across NYHA class groups, stars indicate results of Dunnet’s post hoc test (*p<0.05, **p<0.01; as compared to NYHA I on panel E and NYHA II on panel F). Medians, 25 and 75 percentiles and ranges are indicated on logarithmic scales.

No significant differences for MBL or ficolin-2 concentrations between patients and controls, or across NYHA classes, were observed in either of the cohorts (Figure 1 A–D). However, the patients in both cohorts had markedly lower levels of ficolin-3 as compared to healthy controls (both p<0.001), with gradually decreasing levels according to disease severity (Figure 1 E–F). The difference in ficolin-3 levels remained significant after adjustment for age- and gender (data not shown). The lowest ficolin-3 levels were observed in patients with NYHA class IV. According to the results of Dunnet’s post test, ficolin- 3 levels were significantly lower in patients with NYHA class IV (p<0.01) and class III (p<0.01), as compared to class I patients (Hungarian cohort), whereas in the Norwegian cohort a significant difference was observed between class II and class IV patients (p<0.05). The median concentrations of ficolin-3 across CHF severity classes NYHA II-IV were very similar in the two cohorts: 16.9–15.6–13.3 µg/ml for the Hungarian cohort and 20.3–16.6–13.1 µg/ml for the Norwegian cohort.

### Determinants of Low Ficolin-3 levels

**Figure 2:**
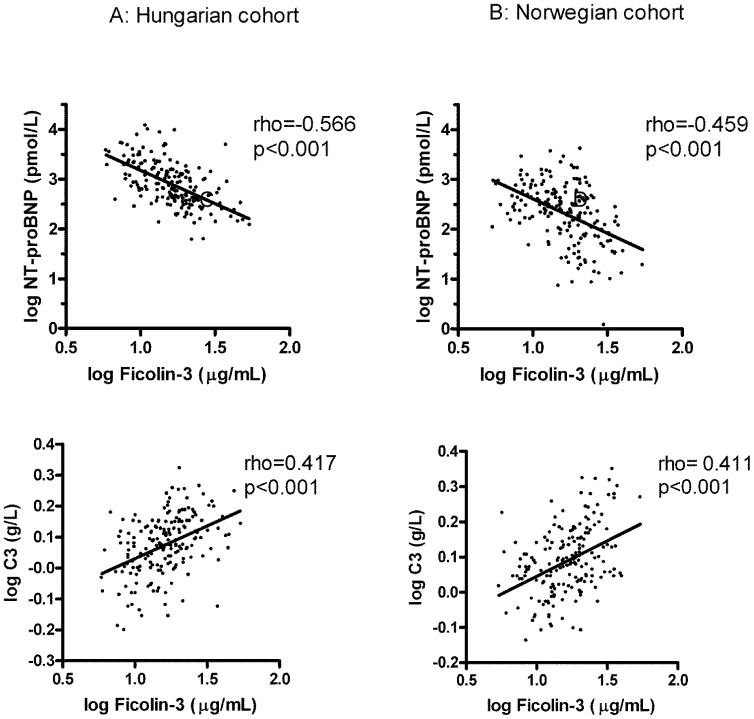
Univariate correlation of ficolin-3 levels with NT-proBNP (A and B) and complement C3 (C and D). Pearson’s correlation coefficients and p values are indicated.

To find the best cut-off level between low and high ficolin-3 levels, a receiver operating characteristic (ROC) analysis of the ficolin-3 concentration in relation to 5 year survival was performed in the Hungarian cohort. The optimal ficolin-3 cut-off level was found to be 15.0 µg/ml. This level was used for further analyses to discriminate between low and high ficolin-3 levels throughout in both cohorts. Using this cut-off level it was revealed that a low ficolin-3 level was associated with decreased body mass index (BMI, p<0.001), low total cholesterol (p<0.001) and increased N-terminal pro-brain natriuretic peptide (NT-proBNP, p<0.001) concentrations in both cohorts. Importantly, no associations with hepatic parenchymal enzymes (i.e., ASAT or ALAT) were observed in either cohort (Table 2). Moreover, no relationship between age or gender, and ficolin-3 levels was observed. Interestingly, we found a significant inverse correlation between ficolin-3 levels and NT-proBNP as a marker of increased myocardial cell wall stress in both cohorts (Figure 2 A–B) (rho = −0.566, p<0.001 and rho = −0.459 p<0.001 for the Hungarian and Norwegian cohorts, respectively). Patients with low ficolin-3 levels were further characterized by low C3 levels in both cohorts (Table 2 and Figure 2 C–D). Again, the shape and strength of associations were very similar in the two cohorts (rho = 0.417, p<0.001 and rho = 0.411, p<0.001 for the Hungarian and Norwegian cohorts, respectively). The plasma levels of the C3 activation marker C3a (measured only in the Hungarian cohort) inversely correlated with ficolin-3 (rho = −0.198, p = 0.008) and high C3a levels were associated with low ficolin 3 concentrations (Table 2). These associations between ficolin-3 and NT-proBNP as well as C3 remained significant after multivariable adjustment (Table S1) for both cohorts.

**Table 2:** Baseline clinical characteristics according to ficolin-3 levels (median, IQ range, or n (%)).

Variable	Hungarian cohort		Norwegian cohort		
Ficolin-3 levels, µg/ml,	<15.0 ug/ml, n = 75	> = 15.0 µg/ml, n = 115	<15.0 mg/L, n = 75		> = 15.0 n = 108
Age	72 (62–78)	67 (58–76)	59 (47–64)		58 (51–64)
Male (%)	51 (68)	89 (77)	58 (78)		85 (79)
Cause of CHF: CAD/IDCM/other	44/19/12 (59/25/16)	64/22/29 (56/19/25)	33/37/4 (45/50/5)		43/59/4 (41/56/4)
NYHA Class I/II/III/IV	7/23/31/14 (9/31/41/19)**	32/38/37/8 (28/33/32/7)	0/13/33/27 (0/18/45/37)*		0/38/46/22 (0/36/43/21)
Hypertension (%)	45 (60)*	85 (74)	9 (12)		18 (16)
T2DM (%)	24 (32)	48 (42)	7 (9)		15 (14)
Previous infarction (%)	31 (41)	45 (39)	25 (34)		37 (34)
LVEF (%)	31 (23–35)***	38 (30–41)	27 (20–36)		30 (22–40)
ACE-I or ARB (%)	50 (67)	79 (69)	65 (87)		90 (83)
Beta blocker (%)	50 (67)	77 (67)	63 (85)		83 (77)
Loop diuretics (%)	58 (77)	83 (72)	58 (78)		72 (67)
Statins (%)	27 (36)	48 (42)	36 (49)		43 (40)
Warfarin (%)	35 (47)	40 (35)	41 (55)*		40 (37)
ASA (%)	24 (32)	48 (42)	26 (35)		52 (48)
BMI (kg/m2)	25.4 (23.7–27.9)***	28.8 (25.0–32.1		24.4 (22.4–27.6)***	27.0 (24.7–31.2)
Creatinine (µM/L)	99 (84–145)	94 (78–118)	99 (83–121)**		84 (71–106)
CRP	6.4 (2.8–15.9)	6.2 (3.1–12.4)	4.3 (2.2–12.0)*		3.2 (1.4–5.6)
NT-proBNP (pM/L)	2351 (794–3081)***	730 (298–883)	376 (218–756)***		142 (48–339)
Total cholesterol mmol/L	3.7 (3.0–4.3) ***	4.4 (3.9–5.2)		3.8 (3.0–4.6)***	4.4 (3.5–5.2)
ASAT (U/L)	27 (19–34)	22 (19–31)		26 (22–32)	27 (22–34)
ALAT (U/L)	23 (16–38)	23 (17–36)		24 (18–36)	25 (20–39)
Hemoglobin (g/L)	141 (124–154)	142 (131–152)		134 (121–149)*	144 (133–150)
Complement C3 (g/L)	1.08 (0.93–1.25)***	1.29 (1.15–1.46)		1.11 (0.95–1.26)***	1.27 (1.12–1.50)
Anaphylatoxin C3a	268.5 (198.7–382.1)	208.8 (120.5–339.3)		n.d.	n.d.

* p<0.050.

** p<0.010.

*** p<0.001;

n.d.: not done.

CAD: coronary artery disease; IDCM: idiopathic dilative cardiomyopathy; NYHA: New York Heart Association; T2DM: Type-2 diabetes mellitus; LVEF: Left-ventricular ejection fraction; CRP: C-reactive protein; NT-proBNP: N-terminal pro-brain natriuretic peptide; ACE-I: angiotensin converting enzyme inhibitor; ASA: Acetyl-salicylic acid; ASAT: aspartate aminotransferase; ALAT: alanin aminotransferase.

### Prediction of 5-year Mortality in CHF by Baseline ficolin-3 levels

**Figure 3:**
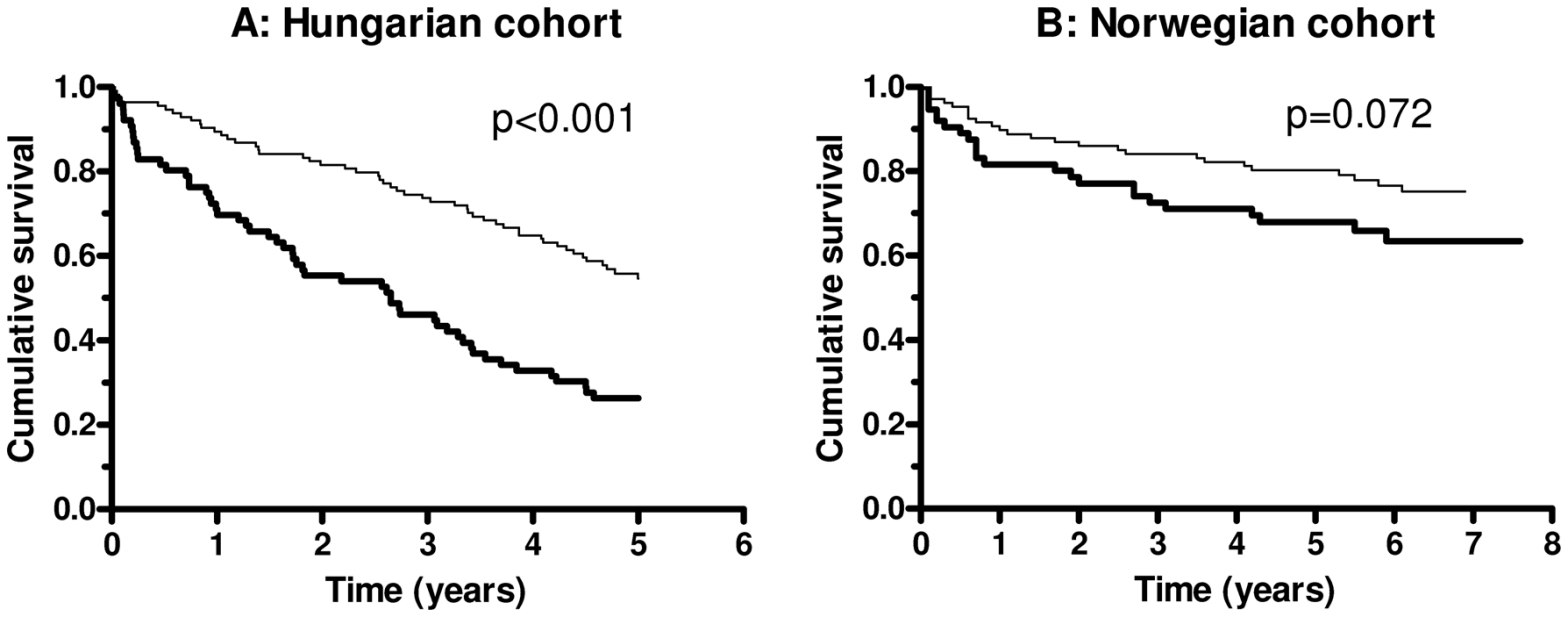
Kaplan-Meier plot of baseline ficolin-3 levels (<15.0 µg/ml, thick line and > = 15.0 µg/ml, thin line) and long term survival (all-cause mortality) in patients with CHF. Log-rank test for the Hungarian cohort (panel A) and for the Norwegian cohort (panel B) is indicated.

During a median follow up of 51 and 67 months (interquartile ranges: 21–60, 32–75, respectively), 107 and 49 patients died in the Hungarian and Norwegian cohorts, respectively (5-year mortality rate 0.56 and 0.27). Ficolin-3 levels were stratified according to the above mentioned ROC analysis and 15.0 µg/ml was determined as cut-point with optimum sensitivity (0.505) and specificity (0.759) to predict all-cause mortality in the Hungarian cohort. Univariate Kaplan-Meier survival curves for patient groups with low or high ficolin-3 levels in the two cohorts, showing an association between low (<15.0 µg/ml) baseline ficolin-3 level and increased all-cause mortality in the Hungarian (p<0.001) and a similar tendency in the Norwegian cohort cohorts (p = 0.072) (Figure 3). Ficolin-3 levels showed significant prediction of mortality in both cohorts in the univariate Cox regression analysis (HR 1.709 and 1.471 per 1-SD decrease of ficolin-3). In multivariable age- and gender adjusted models (Table 3, model 1) low baseline ficolin-3 was associated with a significantly increased risk of mortality in both cohorts. Further adjustment for NT-proBNP (Table 3, model 2) had only minor influence on this association with a significant association of ficolin-3 with total mortality in both cohorts (Table 3). Finally, as shown in Table S2, ficolin-3 remained a significant predictor of mortality in both cohorts if BMI, diabetes mellitus, haemoglobin, or creatinine (one-at-a-time) was added to model 2 of Table 3 (exceptions were haemoglobin and creatinine in the Norwegian cohort, where borderline significance was observed). There were 18 heart transplantations during follow-up in the Norwegian cohort. When low baseline ficolin-3 levels were analyzed in relation to the combined end-point transplantation and mortality, the univariate Kaplan Meier log rank analysis was significant (p = 0.003) (data not shown) and the age-, gender- and NT-proBNP adjusted analysis gave a HR of 1.575 (95% 1.106–2.242) with a Wald’s chi-square of 6.93 (p = 0.008).

**Table 3:** Results of multivariable Cox proportional-hazards regression analyzing effects of ficolin-3 for all-cause mortality.

	HR*	95% CI	?^2^ **	p
**Hungarian cohort**				
Ficolin-3, univariate	1.709	1.317–2.212		<0.001
Ficolin-3, model 1 (adjusted for age and sex)	1.669	1.283–2.169	17.55	<0.001
Ficolin-3, model 2 (adjusted for age, sex and NT-proBNP)	1.368	1.052–1.776	6.210	0.013
**Norwegian cohort**				
Ficolin-3, univariate	1.471	1.106–1.957		0.008
Ficolin-3, model 1 (adjusted for age and sex)	1.457	1.039–2.041	5.194	0.023
Ficolin-3, model 2 (adjusted for age, sex and NT-proBNP)	1.426	1.013–2.008	4.436	0.035

* Hazard ratio for ficolin-3 shown as standardized hazard ratio (HR per 1 SD decrease); age was considered as time-dependent covariate.

** Wald χ^2^ of likelihood-ratio test.

## Discussion

Complement activation has been shown to be an important inflammatory component in many diseases. The molecular mechanisms behind the progressive loss of viable heart tissue in CHF are only partly resolved. In this study we provide evidence, for the first time, that ficolin-3 the main activator of the lectin complement pathway may be associated with advanced heart failure. Firstly, decreasing ficolin-3 concentrations were associated with disease severity in CHF patients as assessed by clinical NYHA classification and strongly inversely correlated with neurohormonal NT-proBNP assessment as a marker of myocardial cell wall stress. Secondly, low ficolin-3 levels were related to high concentrations of C3a levels, combined with decreased C3 concentration, linking ficolin-3 levels with complement activation. Finally, low baseline ficolin-3 levels were associated with 5-year mortality. Importantly these data were consistently obtained in two independent cohorts from two different countries.

The lectin pathway initiator molecules MBL, ficolin-2 and ficolin-3 have all been shown to serve as bridging molecules between apoptotic bodies and phagocytic cells [17]–[20]. For the ligand specificity of ficolin-3 it is known that altered self structures, mainly yet unidentified non-carbohydrate acetylated molecules, are recognized [21]. This interaction is currently best modeled in vitro by the application of acetylated albumin as target molecule. Using this in vitro model system ficolin-3 clearly induces complement activation with deposition of C4, C3 and TCC [22]. Myocytes die by multiple mechanisms in failing hearts, including necrosis, apoptosis, oncosis and autophagy, and these processes are linked with complement activation [23], [24]. A link between complement activation and myocardial failure was further supported by Oliveira et al. showing that increased deposition of TCC in failing myocardium was normalized after prolonged mechanical circulatory support [25]. Compiling these previous observations together with the present ficolin-3 data we suggest that myocardial cell death and altered self structures in the failing human heart may bind ficolin-3, leading to reduced plasma levels and induction of complement activation. The following observations support this conclusion. First, decreased ficolin-3 levels were linked with more severe heart failure, as indicated by significant associations with high NT-proBNP levels and advanced NYHA functional class groups. In addition, ficolin-3 levels showed correlation with complement C3 activation as judged by increased C3a and reduced C3 reflecting consumption. No such correlation was seen for MBL and ficolin-2 (data not shown). A scenario could be that the low ficolin-3 levels are due to consumption because of binding to altered self structures in the myocardial cell wall, which is followed by complement activation, leading to inflammation and tissue damage. In this regard it should be noted that ficolin-3 is by far the most potent activator of the lectin pathway of complement, independent of differences in the circulating levels of the proteins [26].

The majority of complement proteins, including MBL, ficolin-2 and ficolin-3 are produced in the liver and an alternative explanation for the decreased ficolin-3 levels in CHF could be related to a decreased hepatic production and output. Indeed, the synthetic capacity of the liver may be decreased due to hepatic congestion caused by a decreased right ventricular function. We, therefore, investigated whether hepatic congestion could contribute to lower ficolin-3 levels in our patient cohorts. We found, however, that activities of hepatic parenchymal enzymes ASAT and ALAT, markers of liver dysfunction, were not associated with lower ficolin-3 levels making a scenario of decreased synthesis less probable. Furthermore, low levels of MBL and ficolin-2, which both are synthesized in the liver, were not related to CHF severity in our cohorts. These results collectively indicate that the decreased ficolin-3 levels observed in advanced CHF patients were not a result of decreased synthesis by the liver.

It may also be suggested that individuals with low levels of ficolin-3 were predisposed to CHF because of an inherited trait in the *FCN3* gene. Screening of the *FCN3* gene and the promoter have not revealed genomic alterations that may explain inter-individual variation in ficolin-3 plasma concentration as a cause of the observations made in the present study [11] even though a rare deletion in the FCN3 gene may give rise to a novel immunodeficiency syndrome [27].

An increasing number of recent clinical studies have addressed the potential role of ficolin-3 in different inflammatory and in ischemia-reperfusion related conditions. It has been shown that the lectin complement pathway activated by ficolin-3 may be involved in C4d deposition on peritubular capillaries in kidney allografts, and that this process is related to the pathogenesis of humoral rejection [28]. It has also been shown that high pre-transplant levels of ficolin-3 are associated with kidney graft survival [29]. Ficolin-3 appears to be consumed in preeclampsia and binds to apoptotic placentas [30]. Moreover, ficolin-3 has been shown to be decreased in patients with diabetic nephropathy [31], and that it varied significantly between type 2 diabetes patients and controls [32]. Recently, our group reported that concentrations of ficolin-3 were significantly decreased in both the admission and in the follow-up samples of patients with definite ischemic stroke as compared to healthy subjects [33]. An inverse correlation was observed between low ficolin-3 levels and high concentration of S100beta, an indicator of the size of cerebral infarct suggesting that ficolin-3 contributes to the pathogenesis of ischemic stroke. In line with these data our results suggest that ficolin-3 also could be related to the progression of CHF.

It should be noted that the survival rate in the Norwegian cohort is better even though the ratio of patients with a more severe NYHA classification is higher than in the Hungarian cohort. This seemingly paradoxical finding is probably due to the pronounced heterogeneity between the two cohorts (higher age, male predominance and higher proportion of ischemic heart disease in the Hungarian cohort, differences in the origin and genetic background of the two population), but could also reflect underlying differences in the health system between the two countries.

There are particular strengths of this study. We made the observations of low ficolin-3 levels in advanced CHF in two independent cohorts making the problem of type one error mistake less likely. Although there were significant baseline clinical differences between the two cohorts (Table 1), low ficolin-3 were strikingly similar in analytical terms in the different NYHA functional class groups. However, disease severity was assessed by only NYHA functional class and biomarkers, and 6-min walking test was not studied. These observations indicate that disease activity related factors, rather than concomitant clinical confounders, regulate ficolin-3 levels in the two cohorts. Potential limitations of the study are the study design that precluded serial samples from each patient in different stages of the disease course, the number of patients included and the lack of specific causes of death allowing analyzing all-cause mortality only.

In conclusion, our results show that ficolin-3 levels are associated with disease severity in CHF and long term outcome. The inverse correlation with C3a and the correlation with C3 levels link this observation directly to complement activation suggesting that ficolin-3 may be important in aggravation and progression of CHF.

## Materials and Methods

### Hungarian Cohort

One-hundred and ninety consecutive patients with clinical signs of CHF according to the New York Heart Association (NYHA) functional class I-IV and with left ventricular ejection fraction (LVEF) <45% independent of etiology were included in this observational study from the out- or inpatient cardiology departments (Table 1). All of the patients included from the inpatient department were in as compensated fluid state, as it was possible for the given patient, when blood was taken for biomarker determinations (before leaving the hospital). Patients with co-existing malignant or acute infectious conditions were not included. The patients were contacted at year 5 after inclusion and clinical status was registered. Information on mortality (with specific cause of death) was collected from the hospital database, medical records or from family members. Healthy Hungarian controls (n = 100, 54 women; mean±SD age 36±8.9 years) were recruited in an outpatient department in Budapest providing regular health checkup for healthy employees on a mandatory basis.

### Norwegian Cohort

One-hundred and eighty three patients with stable CHF according to NYHA functional class group II-IV independent of etiology were consecutively included in this observational study (Table 1). In the Norwegian sub-study, the actual department of cardiology is a tertiary referral centre for evaluation of patients for heart transplantation or other forms of invasive intervention. All patients were in a stable phase, with no change in medication during the preceding three months. Patients with acute coronary syndromes during the last 6 months and patients with significant concomitant disease, such as infection, malignancy, or autoimmune disease, were excluded. The patients were contacted 4.62 years (mean) after inclusion and all-cause mortality was registered based on the data from the Norwegian National Board of Health Registry. Control subjects were 20 sex- and age-matched healthy individuals from the same area of Norway as the patients (17 men and 3 women; mean±SD age 56±7 years).

### Ethics

Both studies were carried out in accordance with the Helsinki Declaration. The study protocols were approved by the individual Local Ethics Committees: The Institutional Ethics Committee of Semmelweis University in Budapest and The Research Ethics Committee South East under the University of Oslo. Written informed consent approved by the Local Ethical Committees was obtained from all individuals.

### Laboratory Methods

The plasma concentrations of the MBL [34], ficolin-2 [12] and ficolin-3 [11] were determined by established ELISA-based methods. Briefly, microtiter plates were coated with either monoclonal anti-MBL antibody (HYB-131-01) or monoclonal anti-ficolin-2 antibody (FCN216) or monoclonal anti-ficolin-3 antibody (FCN334) in phosphate buffered saline (PBS) overnight at 4°C. Samples diluted 1∶25 and 1∶400 for MBL, 1∶50 for ficolin-2 or 1∶640 for ficolin-3 in sample buffer (PBS-T with 1% mouse serum and bovine serum) were added in triplets to washed wells and incubated for 3 hours at 37°C. MBL was detected with biotinylated monoclonal anti-MBL antibody (HYP-131-01), ficolin-2 was detected with biotinylated monoclonal anti-ficolin-2 antibody (FCN219) and ficolin-3 was detected with biotinylated monoclonal anti-ficolin-3 antibody (FCN334) by incubation overnight at 4°C. Washed wells were incubated for 1 hour at 37°C with HRP-conjugated streptavidin. Plates were developed for 15 minutes with OPD (o-phenylenediamine) substrate solution and stopped by adding 1M H_2_SO_4_. The optical density was measured at 490 nm. As standard was used a serum pool which has been calibrated towards recombinant MBL, ficolin-2 and ficolin-3 and thus contain known concentrations of the different proteins. Lower limit of detection in these assays is 20 ng/ml of MBL, 5 ng/ml of ficolin-2 and 1 ng/ml of ficolin-3, respectively. The inter-assay coefficient of variation (CV) is 8.0%, 7.1% and 4.7% and the intra-assay CV 5%, 4.3% and 3.9% for the MBL, ficolin-2 and ficolin-3 assays, respectively.

For the Hungarian cohort, C3 levels were measured by Roche Cobras Integra 800 (Tina-quant® C3c 2. Cat. No.: 3001938). Levels of C3a (C3a des-arg, Quidel, San Diego, CA, USA), and N-terminal pro-brain natriuretic peptide (NT-proBNP) (Biomedica ELISA kit (Cat No. BI-20852) were determined by commercial ELISA kits according to the manufacturer’s instructions. Other laboratory parameters including C-reactive protein (CRP) were measured by Roche Integra 800, or by Cell-Dyn 3500 hematology analyzer.

For the Norwegian cohort NT-proBNP and CRP levels were assayed on a MODULAR platform (Roche Diagnostics, Basel, Switzerland). C3 levels were determined using nephelometry (BN Prospec, Siemens Healthcare Diagnostics, Deefield, IL) according to the manufacturer’s instructions. Other parameters were measured on a Roche/Hitachi 917 analyzer (Roche Diagnostics, Mannheim, Germany).

Samples were taken at inclusion time. For both cohorts samples were drawn in dry, citrate or EDTA containing vacutainer tubes. All samples were stored at −80°C.

### Statistical Analysis

For descriptive purposes the values of each measurement are given as median and 25^th^–75^th^ percentile, or as numbers (percent), since most of the variables were not normally distributed. Non-parametric tests were used for group comparisons in case of clinical variables (Table 1); continuous variables between two groups were compared with Mann-Whitney U test and with more groups with Kruskall-Wallis test, whereas categorical variables were compared with Pearson’s χ^2^ test. Ficolin-3 showed skewed distribution, therefore, group comparisons were done by parametric ANOVA on log-transformed values. Accordingly, correlation analysis of ficolin-3 and other markers was done by the Pearson’s method on log-transformed variables. Multiple linear regression models with stepwise forward selection process (with an F value >1 to select into the model on log-transformed or categorical variables) was also applied. Kaplan-Meier analysis and log-rank test was used to analyze survival across different strata. Ficolin-3 was fitted to univariate and multivariate Cox proportional hazard model to assess the effect on CHF event free survival. Survival times were measured from inclusion in the study until mortality of any cause. The results of the Cox regression models are presented as hazard ratios (HR) standardized on 1-SD decrease of ficolin-3, the corresponding 95% confidence intervals (CI) and the Wald chi-square and p values of likelihood ratio tests. Age was analyzed as time-dependent covariate in the multivariate models. Receiver operator characteristics (ROC) analysis was applied in the Hungarian cohort to determine the best cut-point of continuous variables to predict clinical events, which also were applied to the Norwegian cohort. Statistical analyses were carried out using the software STATISTICA 7.0 (StatSoft Inc., Tulsa, OK, USA), SPSS 13.01 (Apache Software Foundation, USA) and GraphPad Prism 4.03 (GraphPad Softwares Inc, CA, USA) softwares. Two tailed p values were calculated.

## Supporting Information

Table S1 **Multiple linear regression showing associations between ficolin-3 and baseline clinical and laboratory parameters in patients with chronic heart failure.**
(DOC)

Table S2 **Results of multivariable Cox proportional-hazards regression analyzing effects of ficolin-3 for all-cause mortality.**
(DOC)
