# Supplementary material for: Association of Ficolin-3 with Severity and Outcome of Chronic Heart Failure
Source: PLoS One. 2013 Apr 15;8(4):e60976. doi: 10.1371/journal.pone.0060976 (PMC3626638; doi:10.1371/journal.pone.0060976)
Supplement: Table S1 — Multiple linear regression showing associations between ficolin-3 and baseline clinical and laboratory parameters in patients with chronic heart failure. (DOC) [file pone.0060976.s001.doc]

**Supplemental table S1.** Multiple linear regression showing associations between ficolin-3 and baseline clinical and laboratory parameters in patients with chronic heart failure.

| **Variable** | **Coefficient** | **Standard error** | **t** | **p** |
| --- | --- | --- | --- | --- |
| **Hungarian cohort** | | | | |
| NT-proBNP | -0.186 | 0.025 | -7.337 | 0.000 |
| Total cholesterol | 0.215 | 0.099 | 2.174 | 0.031 |
| Hypertension | 0.068 | 0.023 | 2.946 | 0.004 |
| Complement C3a | -0.119 | 0.037 | -3.232 | 0.001 |
| Complement C3 | 0.326 | 0.141 | 2.309 | 0.022 |
| **Norwegian cohort** | | | | |
| NT-proBNP | -0.133 | 0.034 | -3.926 | 0.000 |
| Complement C3 | 0.282 | 0.060 | 4.714 | 0.000 |
| ACE-I or angiotensin receptor blockers | -0.127 | 0.043 | -2.977 | 0.004 |
| Hemoglobin | 0.879 | 0.361 | 2.437 | 0.016 |
| Gender | -0.089 | 0.043 | -2.100 | 0.038 |
| Hypertension | 0.100 | 0.048 | 2.062 | 0.041 |

Variables presented in Table 2 were analyzed as independent variables by forward selection method.
