# Supplementary material for: Association of Ficolin-3 with Severity and Outcome of Chronic Heart Failure
Source: PLoS One. 2013 Apr 15;8(4):e60976. doi: 10.1371/journal.pone.0060976 (PMC3626638; doi:10.1371/journal.pone.0060976)
Supplement: Table S2 — Results of multivariable Cox proportional-hazards regression analyzing effects of ficolin-3 for all-cause mortality. (DOC) [file pone.0060976.s002.doc]

**Supplemental table S2.** Results of multivariable Cox proportional-hazards regression analyzing effects of ficolin-3 for all-cause mortality

|  | HR* | 95% CI | χ^2^** | p |
| --- | --- | --- | --- | --- |
| **Hungarian cohort** |  |  |  |  |
| Ficolin-3, model 2 in Table 2, plus BMI | 1.346 | 1.033-1.751 | 5.438 | 0.020 |
| Ficolin-3, model 2 in Table 2, plus Diabetes mellitus | 1.389 | 1.060-1.818 | 6.467 | 0.011 |
| Ficolin-3, model 2 in Table 2, plus creatinine | 1.486 | 1.124-1.953 | 8.938 | 0.003 |
| Ficolin-3, model 2 in Table 2, plus hemoglobin | 1.351 | 1.037-1.761 | 5.560 | 0.018 |
| **Norwegian cohort** |  |  |  |  |
| Ficolin-3, model 2 in Table 2, plus BMI | 1.572 | 1.019-2.028 | 4.522 | 0.033 |
| Ficolin-3, model 2 in Table 2, plus diabetes mellitus | 1.437 | 1.027-2.012 | 4.773 | 0.029 |
| Ficolin-3, model 2 in Table 2, plus creatinine | 1.374 | 0.980 -1.923 | 3.594 | 0.058 |
| Ficolin-3, model 2 in Table 2, plus hemoglobin | 1.368 | 0.973 -1.923 | 3.449 | 0.063 |

* Hazard ratio for ficolin-3 shown as standardized hazard ratio (HR per 1 SD decrease).

** Wald χ^2^ of likelihood-ratio test.

Model 2: adjusted for age, gender, NT-proBNP (age was considered as time-dependent covariate).
